# Platinum Nanoparticles Modified Copper/Titanium Electrodes as Electrocatalysts for Borohydride Oxidation

**DOI:** 10.3390/ma14247663

**Published:** 2021-12-12

**Authors:** Aldona Balčiūnaitė, Aušrinė Zabielaitė, Daina Upskuvienė, Loreta Tamašauskaitė-Tamašiūnaitė, Irena Stalnionienė, Leonas Naruškevičius, Jūratė Vaičiūnienė, Algirdas Selskis, Remigijus Juškėnas, Eugenijus Norkus

**Affiliations:** State Research Institute, Center for Physical Sciences and Technology (FTMC), Saulėtekio Ave. 3, LT-10257 Vilnius, Lithuania; aldona.balciunaite@ftmc.lt (A.B.); ausrine.zabielaite@ftmc.lt (A.Z.); daina.upskuviene@ftmc.lt (D.U.); irena.stalnioniene@ftmc.lt (I.S.); leonas.naruskevicius@ftmc.lt (L.N.); jurate.vaiciuniene@ftmc.lt (J.V.); algirdas.selskis@ftmc.lt (A.S.); remigijus.juskenas@ftmc.lt (R.J.); eugenijus.norkus@ftmc.lt (E.N.)

**Keywords:** platinum, copper, titanium, electroless deposition, galvanic displacement, borohydride, oxidation

## Abstract

In this study, sodium borohydride oxidation has been investigated on the platinum nanoparticles modified copper/titanium catalysts (PtNPsCu/Ti), which were fabricated by employing the electroless copper plating and galvanic displacement technique. ICP-OES, XRD, FESEM, and EDX have been used to characterize PtNPsCu/Ti catalysts’ composition, structure, and surface morphology. The oxidation of sodium borohydride was examined on the PtNPsCu/Ti catalysts using cyclic voltammetry and chrono-techniques.

## 1. Introduction

Direct borohydride fuel cells (DBFCs) became the subject of much current research once again, despite the high price of borohydrides manifesting themselves either as alternative hydrogen sources or anodic fuels [1,2,3]. DBFCs have the theoretical voltage of 1.64 V, which is much higher in comparison with other fuel cells, e.g., hydrogen (1.24 V) or methanol (1.19 V) [1,2,3]. Moreover, DBFCs have a high theoretical specific energy density of 9.3 Wh/g and capacity of 5.6 Ah/g for NaBH_4_. Another significant future is power generation at low temperatures. However, these favorable properties have still not been fully implemented in practice. The implementation of DBFCs is still restricted due to the anodic reactions kinetics and insufficient catalytic stability. Active search for various anode catalysts able to realize a kinetics maximum of eight electrons oxidation of sodium borohydride (BOR) is still in progress. Operation of DBFCs is based on the BOR with eight electrons at the anode (Equation (1)) and the O_2_ reduction at the cathode (Equation (2)) and can be presented by the following equations:

BH_4_^−^ + 8OH^−^ → BO_2_^−^ + 6H_2_O + 8e^−^  *E*_0_ = −1.24 V vs. SHE (1)

2O_2_ + 4H_2_O + 8e^−^ → 8OH^−^   *E*_0_ = 0.40 V vs. SHE (2)

The final reaction can be presented by this equation:

BH_4_^−^ + 2O_2_ → BO_2_^−^ + 2H_2_O   *E*_0_ = 1.64 V vs. SHE (3)

It is well-known that hydrolysis of sodium borohydride coincides with the BOR generating hydroxyl borohydride intermediate and hydrogen in various steps [4,5,6,7,8,9,10,11,12,13,14,15]:

BH_4_^−^ + H_2_O → BH_3_(OH)^−^ + H_2_ (4)

BH_3_(OH)^−^ + H_2_O → BO_2_^−^ + 3H_2_ (5)

where the overall reaction is as follows:

BH_4_^−^ + H_2_O → BO_2_^−^ + 4H_2_ (6)

This phenomenon depends on the electrode material. In this case, on nickel [5,9,10,16,17] and Pt [10,15], the final reaction of borohydride oxidation occurs through a four-electron process:

BH_4_^−^ + 4OH^−^ → BO_2_^−^ + 2H_2_O + 2H_2_ + 4e^−^ (7)

However, BH_4_^−^ hydrolysis yielding H_2_ reduces DBFC coulombic efficiencies (as direct oxidation of BH_4_^−^ might not take place) and has negative effects on the catalytic reaction active area. 

Platinum is usually accepted as the only single component catalyst having the best catalytic activity and is the most frequently used catalyst for the BOR despite its high cost. The Pt-based catalysts with transition metals (Ni, Co, Fe, Cu) have been developed to reduce the amount of Pt in the catalysts. The resulting catalysts exhibited higher activity and better catalytic stability for the BOR, methanol oxidation, and oxygen reduction compared with pure Pt catalyst [6,7,8,9,10,11,12,13,14,15,18,19,20,21,22,23,24,25,26,27,28]. 

One more opportunity to create effective anode materials for DBFCs is to look for proper support. Nowadays, different carbon materials are the most widely used support for Pt catalysts allowing to minimize the utilization of noble metals. Still, recently non-carbon materials such as titanium have been accepted as a promising alternative for carbon supports due to its advantages: high chemical and electrochemical stability and good electronic conductivity. The titanium mesh coated with PtRuO_x_, Pt_3_Ti, PtSn/Ti, the platinized Ti electrode, and Pt dispersed on Ti metal catalysts show an improved electrocatalytic activity for methanol electro-oxidation [29,30,31,32,33,34]. The authors in Ref. [35] evaluated the enhanced electrocatalytic activity of carbon-supported PtTi alloy for the oxygen reduction reaction.

In the study presented herein, well-adherent platinum nanoparticles modified copper films (denoted as PtNPsCu/Ti), which have Pt particle size in a few nanometers, were prepared using low-cost and straightforward electroless Cu deposition and galvanic displacement techniques [20,36,37,38,39,40]. The activity of the as-prepared PtNPsCu/Ti catalysts for BOR in an alkaline solution was examined using cyclic voltammetry (CV) and chrono-techniques (chronoamperometry (CA) and chronopotentiometry (CP)). Inductively Coupled Plasma Optical Emission Spectrometry (ICP-OES), X-ray diffraction (XRD), Field-Emission Scanning Electron Microscopy (FESEM), and Energy Dispersive X-ray Spectroscopy (EDX) were applied for the samples’ composition, structure, and surface morphology characterization. 

## 2. Materials and Methods

### 2.1. Chemicals

NaBH_4_, CoCl_2_, CuCl_2_, HCl, diethylenetriamine, H_2_PtCl_6_, and titanium foil of 0.127 mm thick and 99.7% purity were received from Sigma-Aldrich Supply (Darmstadt, Germany). H_2_SO_4_ (96%), ethanol, and NaOH (99%) were obtained from Chempur Company (Karlsruhe, Germany). All chemicals were of analytical grade. Deionized water with a resistivity of 18.2 MΩ cm^−1^ was used to prepare all the solutions.

### 2.2. Preparation of Catalysts

A sublayer of copper was deposited on the titanium according to the procedure described in [41]. After that, the Cu/Ti catalysts were dipped in a 1 mM H_2_PtCl_6_ + 0.1 M HCl solution at room temperature for 5, 15, and 30 min. The surface-to-volume ratio was 1.3 dm^2^ L^−1^. The obtained catalysts were thoroughly rinsed with deionized water and dried in air at room temperature. The BOR was investigated on the prepared catalysts without any further processing.

### 2.3. Catalysts Characterization

An SEM/FIB workstation Helios Nanolab 650 with a dispersive energy X-ray (EDX) spectrometer INCA Energy 350 X-Max 20 (FEI, Eindhoven, The Netherlands) was used to examine the morphology and composition of the fabricated catalyst.

XRD patterns of PtNPsCu/Ti and Cu/Ti were estimated employing an X-ray diffractometer SmartLab (Rigaku, Japan) equipped with a 9 kW X-ray tube with a rotating Cu anode. The grazing incidence (GIXRD) method was used in the 2Θ range 35–52°. The angle α between the parallel beam of X-rays and the specimen surface (ω angle) was adjusted to 0.5°.

The Pt loading was determined using an ICP optical emission spectrometer Optima 7000DV (Perkin Elmer, Waltham, MA, USA). 

### 2.4. Electrochemical Measurements

A potentiostat PGSTAT100 (Metrohm Autolab B. V., Utrecht, The Netherlands) was employed for all electrochemical measurements using an electrochemical cell, where Cu/Ti, PtNPsCu/Ti, and pure Pt catalysts were applied as working electrodes. An Ag/AgCl/KCl (3 M KCl) and a Pt foil were employed as reference and counter electrodes. The measured current densities were normalized concerning the geometric area of catalysts equal to 2 cm^2^. 

Cyclic voltammograms (CVs) were recorded in a 1 M NaOH solution and in a 1 M NaOH solution containing 0.05 M NaBH_4_ at a temperature of 25 °C at a potential sweep rate of 10 mV s^−1^. The electrochemically active areas (ESAs) of platinum surface in the catalysts were obtained from the CVs of pure Pt and PtNPsCu/Ti catalysts recorded in an Ar-deaerated 0.5 M H_2_SO_4_ solution at a sweep rate of 50 mV s^−1^ by calculating the charge associated with hydrogen adsorption (220 µC cm^−2^) [42].

The CA experiments were carried out by, at first, holding the potential at the open circuit for 10 s and then stepping to −0.7 and 0.1 V for 120 s, respectively. 

CP curves were recorded at a constant current density of 10 mA cm^−2^ vs. the geometric areas of the investigated catalysts for 120 s.

### 2.5. Investigation of the Catalytic Hydrolysis of NaBH_4_

A classic water-displacement method was used to determine the volume of generated H_2_ catalyzed by the PtNPsCu/Ti catalyst. The catalyst was prepared by immersing Cu/Ti in 1 mM H_2_PtCl_6_ + 0.1 M HCl at room temperature for 15 min. In a typical measurement, the NaBH_4_ and NaOH solution was thermostated in an airtight flask fitted with an outlet to collect generated H_2_ gas. Then PtNPsCu/Ti was immersed into a solution with different temperatures (40, 50, 60, and 70 °C) to initiate a hydrolysis reaction. The water displaced from a graduate cylinder connected to the reaction flask was continually monitored as the reaction proceeded. 

## 3. Results

**Figure 1:**
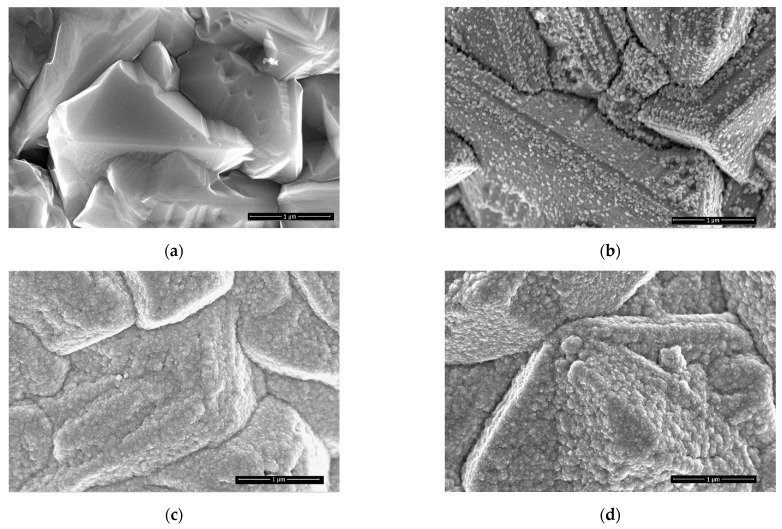
Topside views of: (**a**) Cu/Ti; (**b**–**d**) PtNPsCu/Ti. The deposition time of PtNPs was 5 (**b**), 15 (**c**), and 30 (**d**) min.

PtNPs/Cu catalysts with Pt particles of a few nanometers in size were prepared by immersing Cu/Ti electrode into a 1 mM H2PtCl6 + 0.1 M HCl solution for various periods as shown in Figure 1b–d. The Cu sublayer with a thickness of about 1.5 µm and with an average size of crystallites of ca. 1 µm was deposited onto the Ti surface (Figure 1a). Dipping Cu/Ti electrodes into a 1 mM H_2_PtCl_6_ + 0.1 M HCl solution for 5, 15, and 30 min results in the deposition of Pt nanoparticles on the Cu surface. As evident from Figure 1b–d, PtNPs are homogeneously dispersed on the Cu surface and appear as bright crystallites of the cubic shape of 10 up to 50 nm in size.

EDX analysis confirms the presence of Pt and Cu in the prepared catalysts. The data are shown in Table 1. Large amounts of deposited Cu and much lower ones of Pt were determined.

**Figure 2:**
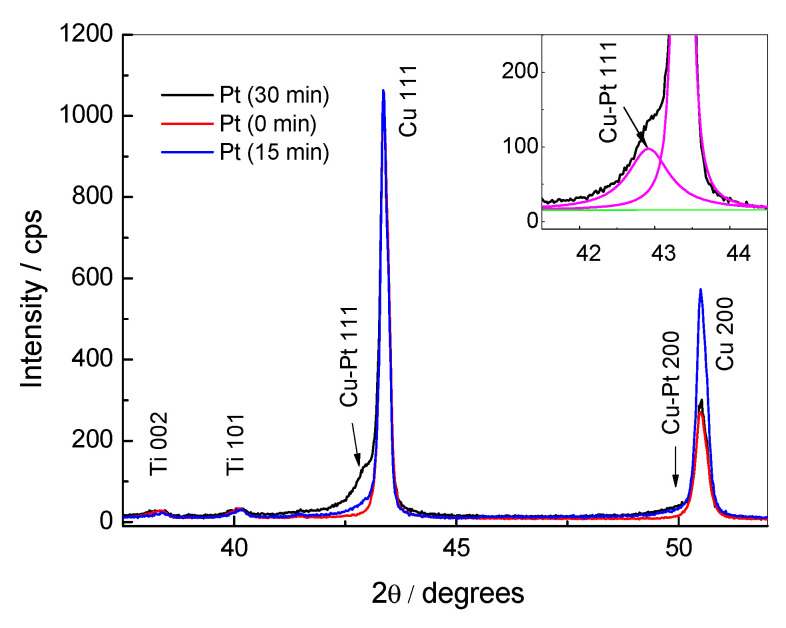
XRD patterns of Cu/Ti (lower red colour pattern) and PtNPsCu/Ti catalysts.

XRD patterns for Cu/Ti (lower curve) and PtNPsCu/Ti prepared by immersing of as-prepared Cu/Ti in 1 mM H_2_PtCl_6_ + 0.1 M HCl for 15 and 30 min (upper curves) are presented in Figure 2. The XRD peaks 100 and 002 of the Ti substrate and strong peaks 111 and 200 of Cu in the case of Cu/Ti were observed. When the Pt crystallites were deposited on the Cu/Ti surface by immersing of the latter catalyst into the Pt(IV)-containing solution for 30 or 15 min (topmost (black) and middle (blue) curves, respectively), a hump on a right shoulder of Cu peaks appeared. This means that a small peak is overlapping with that of copper. The mentioned peaks can be separated using special software, as shown in the inset of Figure 2. The maxima of the low-intensity peaks corresponded to positions of peaks 111 and 200 of an fcc crystalline structure with the lattice parameter of 0.365 nm. Presumably, these peaks are of the fcc phase of a solid solution of Pt in Cu.

According to the determined value of the lattice parameter of the Pt-Cu phase and Vegard’s law, the proportion of Pt in the solid solution was calculated. It was equal approximately to 11.5 at.%. The broad XRD peak (FWHM = 0.635° for the peak with a maximum at 42.92°) of the solid solution pointed to a small size of crystallites and/or very thin Pt-Cu phase layer of about 16.3 ± 0.8 nm. Notably, the broadening of the peaks could also be caused by the inhomogeneity of the composition of the Pt-Cu solid solution.

The Pt loadings were determined to be 2.1, 13.6, and 26.5 µg cm^−2^ in the as-prepared catalysts after Cu/Ti was immersed in the 1 mM H_2_PtCl_6_ + 0.1 M HCl solution for 5, 15, and 30 min, respectively.

**Figure 3:**
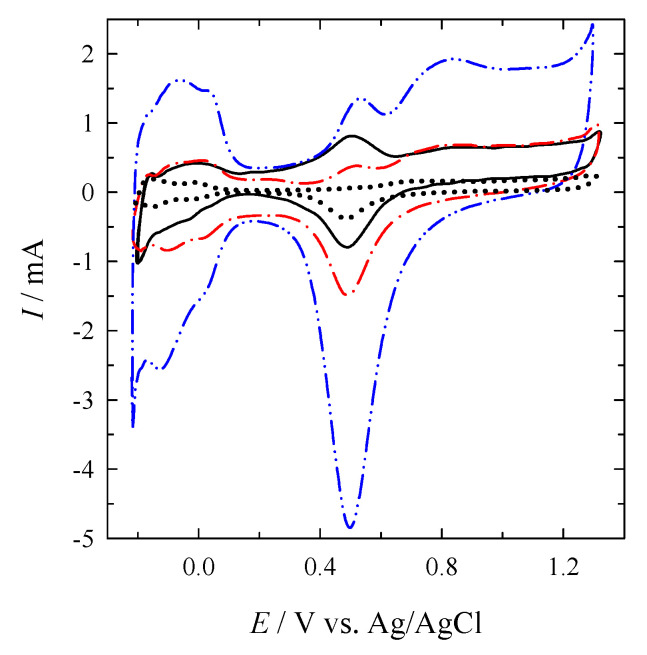
CVs of Pt (*dotted line*) and PtNPsCu/Ti were recorded in Ar-deaerated 0.5 M H_2_SO_4_ at 50 mV s^−1^. The PtNPs deposition time was 5 (*solid line*), 15 (*dash-dotted line*), and 30 (*dash-dot-dotted line*) min.

The electrochemically active surface areas of Pt in the catalysts were determined from the CVs of Pt and PtNPsCu/Ti recorded in an Ar-deaerated 0.5 M H_2_SO_4_ solution in a potential range of −0.2 and 1.3 V at 50 mV s^−1^ (Figure 3). 

The CV profile of PtNPsCu/Ti presents the typical characteristics of the pure Pt electrode as the Ni is electrochemically leached. The obtained ESA value for pure Pt is 2.5 cm^2^, whereas the ESAs values for the PtNPsCu/Ti catalysts obtained after Cu/Ti electrodes immersing in 1 mM H_2_PtCl_6_ + 0.1 M HCl for 5, 15 and 30 min are 8.3, 9.2, and 49.4 cm^2^, respectively. A ca. 3–20 times higher ESAs values of the catalysts prepared by galvanic displacement of Cu layer by PtNPs compared to that of pure Pt were determined. 

**Figure 4:**
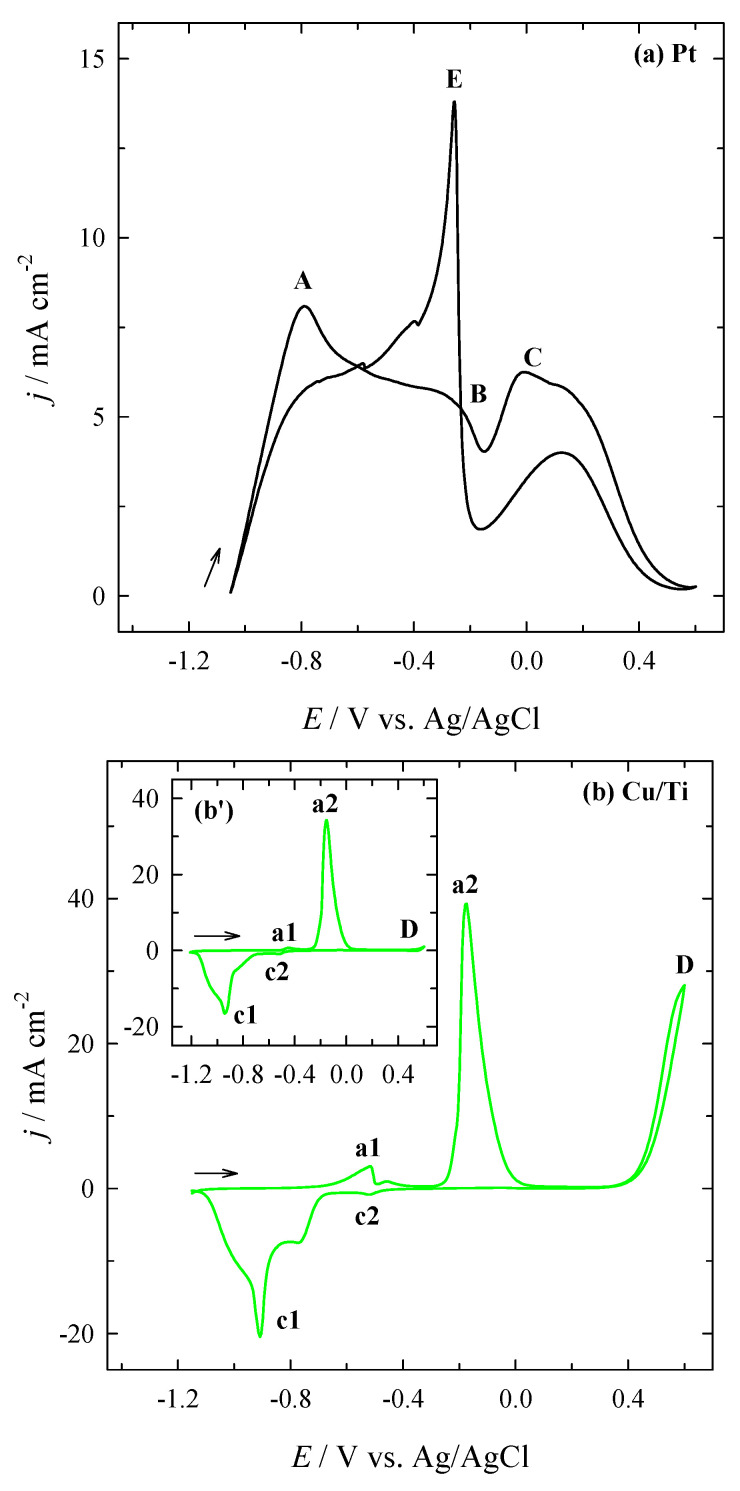
CVs of: (**a**) Pt and (**b**) Cu/Ti recorded in 0.05 M NaBH_4_ + 1 M NaOH at 10 mV s^−1^; 25 °C. The inset b′ presents CV of Cu/Ti in 1 M NaOH.

Figure 4 shows the BOR onto the pure Pt electrode (a) at 10 mV s^−1^. 

As evident, two well-distinguished anodic peaks A and C, are seen in the CV plot. The first peak A is due to the oxidation of H_2_ generated by catalytic hydrolysis of sodium borohydride (Figure 4a). Furthermore, peak B, which is hardly discernible at −0.2 V, could be corresponded to the electro-oxidation of BH_3_OH^−^ according to the following reaction:

BH_3_OH^−^ + 3OH^−^ → BO_2_^−^ + 3/2H_2_ + 2H_2_O + 3e^−^ (8)

At more positive potentials between 0 and 0.2 V, a distinct peak C appears on the Pt electrode (Figure 4a). According to [11,12], this peak and a low wave in the backward scan are associated with the direct BOR, while a sharp asymmetric peak E, that was observed in the backward scan and centered at about −0.3 V, belongs to the oxidation of intermediate BH_3_OH^−^ by Equation (9) that could also be produced in the BOR:

BH_4_^−^ + OH^−^ → BH_3_OH^−^ + H* + e^−^ (9)

The wave detectable at −0.7 V is attributed to BH_3_OH^−^ oxidation which coalesces with the H_2_ oxidation wave.

Figure 4b presents the CVs of Cu/Ti in the background 1 M NaOH solution (the inset b’) and in the 1 M NaOH + 0.05 M NaBH_4_ solution (b) at 10 mV s^−1^. Both CVs for Cu/Ti are similar in shape except those at potential values higher than 0.4 V. Noticeable anodic currents (peak D) were recorded on the latter catalyst in 0.05 M NaBH_4_ + 1 M NaOH. As evident from the CVs of Cu/Ti in the background solution (the inset b’) and 0.05 M NaBH_4_ + 1 M NaOH (b), a lower peak a1 at a potential value of −0.5 V and a larger one a2 at −0.25 V are seen in both CVs plots. According to the literature [43,44], peak a1 corresponds to the formation of insoluble Cu^+^ compounds and peak a2 – to the formation of insoluble Cu^2+^ compounds. The reduction peaks c1 and c2 are seen in the cathodic part during the backward scan. Those peaks may be attributed to reducing the insoluble Cu^+^ and Cu^2+^ compounds formed previously during the anodic scan.

When comparing the CVs for Cu/Ti, recorded in the potential region of −1.2 to 0.6 V in 1 M NaOH solution (Figure 4b′) and 0.05 M NaBH_4_ + 1 M NaOH (Figure 4b), it is seen that the current densities of anodic peaks a1 and a2 are increased. The potential values are slightly shifted to negative potential values (Figure 4b). Observed anodic peaks D at potentials higher than 0.5 V presumably may be attributed to the Cu^3+^/Cu^2+^ couple [44,45,46,47]. This phenomenon always appears due to the oxidation of Cu in strongly alkaline solutions (Figure 4b). The formal reduction potential of Cu^3+^/Cu^2+^ in 1 M NaOH stands at 0.56 V vs. Ag/AgCl [48,49]. Notably, NaBH_4_ is inactive at Cu/Ti at low potentials (Figure 4b). Prominent anodic currents under peak D are observed in the potential region of Cu^2+^/Cu^3+^ transition. They may correspond to the strong interaction of NaBH_4_ with the surface that was already covered by insoluble Cu^2+^ compounds.

**Figure 5:**
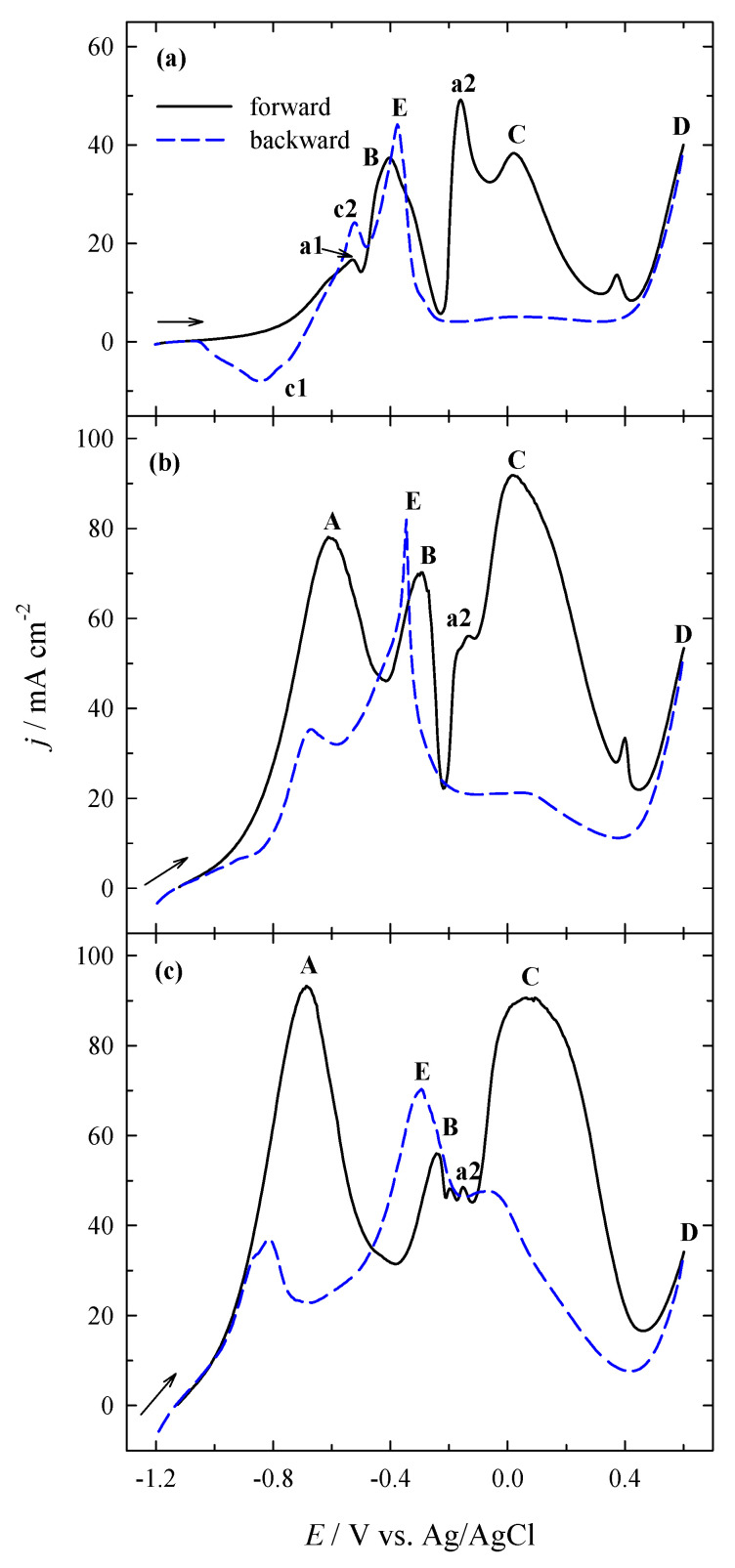
CVs of PtNPsCu/Ti with the Pt loadings of 2.1 (**a**), 13.6 (**b**) and 26.5 (**c**) µg_Pt_ cm^−2^ were recorded in 0.05 M NaBH_4_ + 1 M NaOH at 10 mV s^−1^; 25 °C.

Figure 5 presents BH_4_^−^ ions oxidation on PtNPsCu/Ti that has the different Pt loadings. The BOR proceeds more complicated on the latter catalysts, as evident from the data in Figure 5. Contrary to the bare Pt electrode (Figure 4a), several anodic peaks are seen in the CVs plots, and the shape of CVs depends on the deposited Pt loadings on the Cu/Ti surface. When the Pt loading is 2.1 µg cm^−2^ (Figure 5a), several oxidation peaks are seen on the PtNPsCu/Ti catalyst. Anodic peaks a1 and a2 may correspond to the open sites of Cu crystallites oxidation (Figure 1b) as they occur under the same potential values compared to the CV of Cu/Ti recorded in an alkaline borohydride solution (Figure 4b). With the increase in the Pt loadings, the shape of CVs remains the same as in the case of bare Pt, except for an enhanced anodic peak B at ca. −0.2 V (Figure 5b,c). Notably, the increase in the Pt loading on Cu/Ti results in a comparatively high rise in the values of current densities of peak C, which shows an enhanced catalytic activity of the surface.

**Figure 6:**
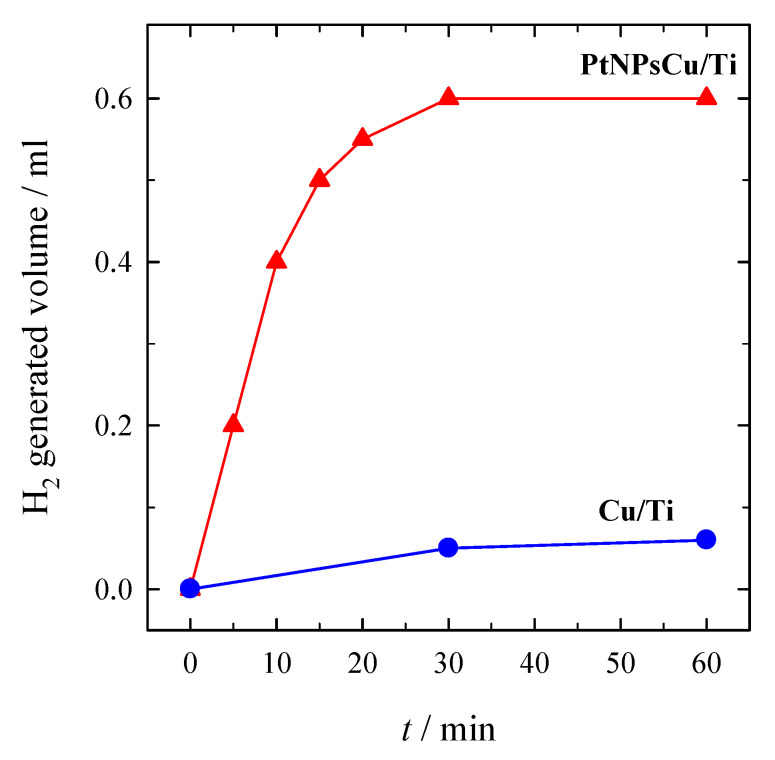
H_2_ generation from 15 mL 0.05 M NaBH_4_ + 1 M NaOH at 25 °C catalyzed by Cu/Ti and PtNPsCu/Ti catalyst with the Pt loading of 13.6 μg_Pt_ cm^−2^.

The same trends are also observed for the oxidation of H_2_ (compare peaks A), which is connected with a higher amount of deposited Pt crystallites on the Cu/Ti surface, as evident in Figure 1c,d. The catalytic activity of Cu/Ti and PtNPsCu/Ti for the hydrolysis of NaBH_4_ was investigated to confirm the nature of anodic peak A. Figure 6 presents data for hydrogen generation catalyzed by Cu/Ti and PtNPsCu/Ti with the Pt loading of 13.6 μg Pt cm^−2^ in the same solution as in Figure 5 at the temperature of 25 °C. A greater hydrogen generation rate was obtained at PtNPsCu/Ti than that at Cu/Ti. This indicates the higher catalytic performance of Pt-Cu alloy for the catalytic NaBH_4_ hydrolysis.

**Figure 7:**
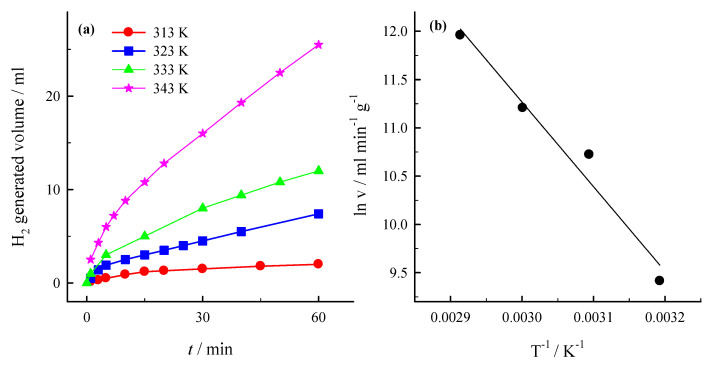
(**a**) Dependence of H_2_ generated volume at PtNPsCu/Ti with the Pt loading of 13.6 µg cm^−2^ on the temperature in 0.264 M NaBH_4_ + 1 M NaOH. T (K): 1–313, 2–323, 3–333 and 4–343. v = 15 mL. (**b**) The Arrhenius plot was calculated from the NaBH_4_ hydrolysis rates in the same solution.

The kinetics of NaBH_4_ hydrolysis was further examined at different temperatures. Figure 7a presents the hydrogen generation rate, which was measured during the hydrolysis of alkaline 0.264 M NaBH_4_ + 1 M NaOH solution using PtNPsCu/Ti with the Pt loading of 13.6 µg cm^−2^ as a function of reaction temperature (40–70 °C).

It was found that the rate of catalytic hydrolysis of sodium borohydride in alkaline solutions increases exponentially with the increase in reaction temperature (Figure 7a). The Arrhenius equation expresses temperature dependence of the rate of hydrogen generation:

(10) $$k=Ae^{-E_{a}/RT},$$

where *E_a_* is the activation energy (J), A-the frequency factor, R-the general gas constant (8.314 J mol^−1^ K^−1^). The activation energy was found from the Arrhenius plot of ln(*k*) vs. 1/*T* (Figure 7b) plotted from the data presented in Figure 7a. The Arrhenius plot gives the energy of 70 kJ mol^−1^. The obtained data confirm that the PtNPsCu/Ti catalyst catalyzes the NaBH_4_ hydrolysis reaction in alkaline solutions.

**Figure 8:**
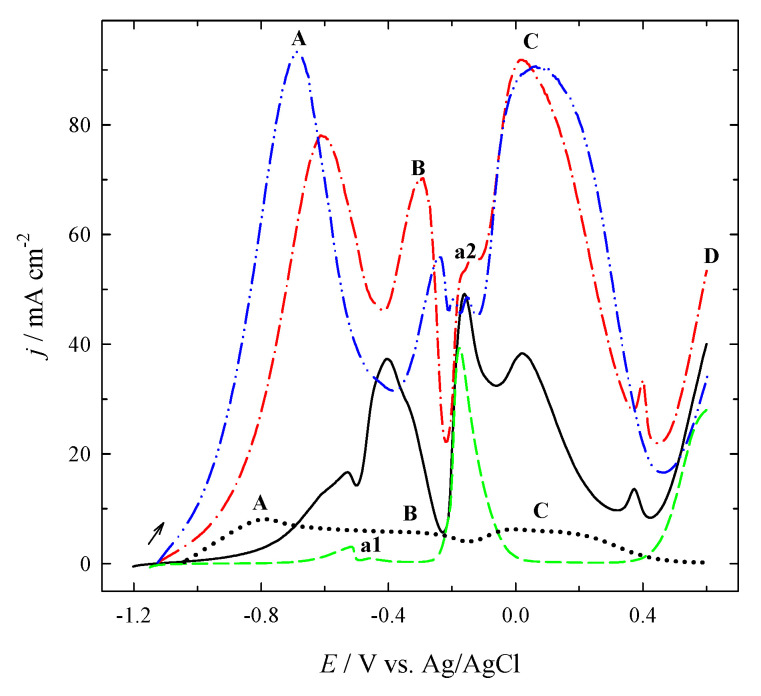
Positive-potential going voltammograms recorded on pure Pt (*dotted line*), Cu/Ti (*dashed line*) and PtNPsCu/Ti catalysts, which have Pt loadings of 2.1 (*solid line*), 13.6 (*dash-dotted line*) and 26.5 (*dash-dot-dotted line*) µg_Pt_ cm^−2^ in 0.05 M NaBH_4_ + 1 M NaOH at 10 mV s^−1^; 25 °C.

The comparison of the positive-potential going voltammograms of BOR recorded on pure Pt (*dotted line*), Cu/Ti (*dashed line*), and different PtNPsCu/Ti catalysts, which have the Pt loadings of 2.1 (*solid line*), 13.6 (*dash-dotted line*) and 26.5 (*dash-dot-dotted line*) µg_Pt_ cm^−2^ at 10 mV s^−1^ is presented in Figure 8. 

The shape of voltammograms for PtNPsCu/Ti is similar to the one recorded on pure Pt, except for the enhanced anodic currents. As depicted from Figure 8, anodic peak A seen in voltammogram for Pt (*dotted line*) and PtNPsCu/Ti catalysts, which have the Pt loadings of 2.1 (*solid line*), 13.6 (*dash-dotted line*), and 26.5 (*dash-dot-dotted line*) µg_Pt_ cm^−2^, is related to the oxidation of H_2_ (HOR) generated by catalytic hydrolysis of sodium borohydride [11]. The HOR is more pronounced on the nanostructured catalysts with more significant Pt loadings. The peak current density values recorded on the catalysts, which have higher Pt loadings of 13.6 and 26.5 µg_Pt_ cm^−2^, are ca. 10–11 times higher than that of bare Pt, while the potential values of peak A are slightly shifted to the side of positive potential values. In the case of PtNPsCu/Ti catalyst with the lower Pt loading of 2.1 µg_Pt_ cm^−2^ (Figure 8, *solid line*), the current density values of the peak A are ca. 2 times higher as compared to that of bare Pt, and is shifted to more positive potential values by 0.3 V. Anodic peaks C seen in the voltammograms are attributed to the direct BOR [11] and also are ca. 6–14 times greater on the PtNPsCu/Ti catalysts as compared to that of bare Pt (Figure 8). The peak B attributed to the electro-oxidation of BH_3_OH^−^, which is hardly discernible at −0.3 V on pure Pt (Figure 8, *dotted line*), is also enhanced on the different PtNPsCu/Ti catalysts. Assuming ca. 3.3 and 3.7 times greater active surface area of PtNPsCu/Ti catalysts, which have the Pt loadings of 2.1 and 13.6 µg_Pt_ cm^−2^ compared to smooth polycrystalline Pt, the surface area normalized current density values of the peaks B and C are ca. 1.8–3.4 and 2.2–4.0, respectively, times higher on those catalysts. Therefore, assuming a ca. 20 times greater ESA of the nanostructured catalyst that has the Pt loading of 26.5 µg_Pt_ cm^−2^ as compared to that of pure Pt, the surface area normalized oxidation currents of the peaks A, B and C are ca. 1.7, 2.0 and 1.4, respectively, times higher on Pt.

Increased electrocatalytic activity of PtNPsCu/Ti may be related to the formation of Pt-Cu alloy and the change of Pt electronic structure due to the presence of Cu [21,50,51,52,53,54,55,56,57]. 

**Figure 9:**
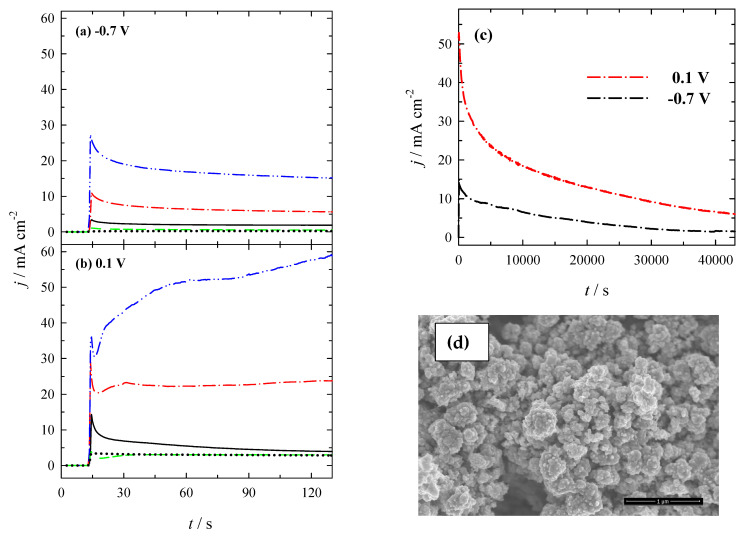
Chronoamperometric data from pure Pt (*dotted line*) Cu/Ti (*dashed line*) and PtNPsCu/Ti catalysts which have Pt loadings of 2.1 (*solid line*), 13.6 (*dash-dotted line*), and 26.5 (*dash-dot-dotted line*) µg_Pt_ cm^−2^ recorded at −0.7 (**a**) and 0.1 (**b**) V in 0.05 M NaBH_4_ + 1 M NaOH. The potential was firstly held at open circuit for 10 s, then set to −0.7 and 0.1 V each for 2 min. (**c**,**d**) represents CAs for PtNPsCu/Ti catalyst (13.6 µg_Pt_ cm^−2^) recorded at −0.7 and 0.1 V for 12 h and the corresponding SEM image of catalyst.

The PtNPsCu/Ti catalyst’s performance was investigated for both processes: HOR (peak A) and BOR (peak C) using chronoamperometry and compared to that of Pt. The corresponding CA curves are shown in Figure 9. 

PtNPsCu/Ti catalysts which have the Pt loadings in the range from 2.1 to 26.5 µg_Pt_ cm^−^^2^ and pure Pt show a current drop-off for the HOR generated by catalytic hydrolysis of NaBH_4_ (Figure 9a). It is seen that at the end of the experimental period (*t* = 130 s), the current densities recorded at PtNPsCu/Ti are more significant, and the current density decay is much slower than that on Pt. Current densities are ca. 7, 22, and 59 times greater at PtNPsCu/Ti catalysts prepared by immersion of Cu/Ti in the 1 mM H_2_PtCl_6_ + 0.1 M HCl solution for 5, 15, and 30 min, respectively, due to oxidation of hydrogen (Figure 9a), whereas active surface area normalized currents are ca. 6, 16, and 3 higher at the PtNPsCu/Ti catalysts than those on Pt. 

Moreover, the PtNPsCu/Ti catalysts, which have a Pt loading of 2.1 and 13.6 µg_Pt_ cm^−^^2^, also show the decay of current densities with reaching constant values within 30–60 s during the oxidation of borohydride (Figure 9b). The latter catalysts have a greater electrocatalytic activity and stability for BOR compared to pure Pt and Cu/Ti electrodes. Ca. 1.4 and 8.4 times higher current densities were obtained at these catalysts due to BOR than those on Pt, whereas active surface area normalized current densities are ca. 1 and 6 times higher at the latter catalysts than those on Pt (Figure 9b). 

Notably, the PtNPsCu/Ti catalyst prepared with a higher Pt loading of 26.5 µg_Pt_ cm^−^^2^ shows a current increase during the BOR (Figure 9b). It may be related to the two consecutive electron transfer steps for the oxidation of PtNPs and Cu at more positive potentials [58,59], indicating the instability of this catalyst.

The long-term chronoamperometric test was also carried out for PtNPsCu/Ti catalyst with Pt loading of 13.6 µg_Pt_ cm^−^^2^ at −0.7 and 0.1 V, respectively, up to 12 h (Figure 9c). As evident, the current densities are decreasing in time, indicating that the catalyst is not sufficiently stable during the long-term test. After 12 h, the final current densities on PtNPsCu/Ti catalyst are 1.6 and 6.1 mA cm^−2^ at −0.7 and 0.1 V, respectively (Figure 9c). Not sufficient catalyst stability can be related to the simultaneously occurring anodic oxidation of Cu underlayer with PtNPs at higher potential values (Figure 9c, red line). Comparison of the initial catalyst surface (Figure 1c) with the surface obtained after long-term chronoamperometric test shows some effects of dissolution of Cu underlayer (Figure 9d).

**Figure 10:**
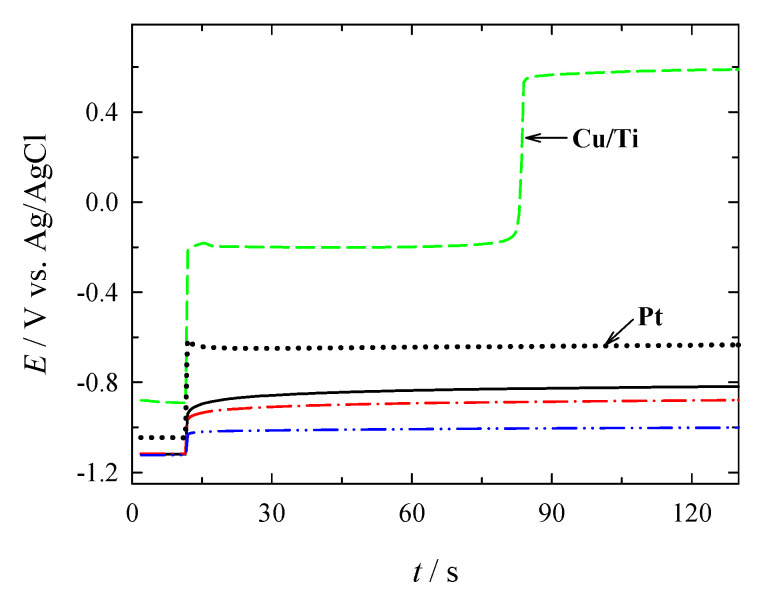
Chronopotentiometric data for bare Pt (*solid line*), Cu/Ti (*dotted line*), and different PtNPsCu/Ti catalysts as in Figure 8. The current step was 0 to 10 mA cm^−2^.

Chronopotentiometry of BOR was also carried out on the PtNPsCu/Ti catalysts. Following a rest period of 10 s at an open circuit, a current density step of 10 mA cm^−2^ was applied to the investigated catalysts for 120 s. The anode potentials (including the open-circuit values between −0.879 and −1.123 V vs. Ag/AgCl) for the catalysts operating at 10 mA cm^−2^ is given in Figure 10.

It was found that the difference between the steady-state operating anode potential and the one at open-circuit was the smallest one on PtNPsCu/Ti with the Pt loading of 26.5 µg_Pt_ cm^−2^. It was equal to ca. 0.122 V, followed by the catalysts, which have the Pt loadings of 13.6 and 2.1 µgPt cm^−2^, where they reached about 0.238 and 0.301 V, respectively. Finally, Pt reached about 0.412 V. Moreover, all the catalysts outperformed Pt under the employed chronopotentiometry conditions.

The as-prepared PtNPsCu/Ti catalysts demonstrated their significantly greater electrocatalytic activity for H_2_ and sodium borohydride oxidation than pure Pt and Cu/Ti. Those catalysts seem to be a promising anodic material for DBFCs.

## 4. Conclusions

A series of PtNPsCu/Ti catalysts have been prepared via galvanic displacement reactions between hierarchical electroless Cu layer and solution of H_2_PtCl_6_. The as-prepared PtNPsCu/Ti catalysts exhibited higher electrocatalytic activity for the oxidation of H_2_, which was generated by sodium borohydride catalytic hydrolysis and sodium borohydride oxidation than that of a bare Pt electrode. The current densities for H_2_ and direct sodium borohydride oxidation are substantially enhanced on the PtNPsCu/Ti catalysts. The prepared PtNPs modified Cu/Ti catalysts seem to be a promising anodic material in producing direct or indirect DBFCs.

## Figures and Tables

**Table 1:** The composition of the same PtNPsCu/Ti catalysts as in Figure 1b–d.

Catalyst	Element, at.%				Pt loading, µg cm^−2^
	Pt	Cu	O	Ti	
b	0.21	94.70	4.32	0.76	2.10
c	1.37	91.90	5.75	0.98	13.60
d	2.92	89.49	6.62	0.97	26.50

## References

[1] Santos D.M.F., Sequeira C.A.C. (2011). Sodium borohydride as a fuel for the future. Renew. Sustain. Energy Rev..

[2] Demirci U.B., Miele P., Garin F. (2011). Catalysis in hydrolysis of sodium borohydride and ammonia borane, and electrocatalysis in oxidation of sodium borohydride. Catal. Today.

[3] Wee J.H.J. (2006). A comparison of sodium borohydride as a fuel for proton exchange membrane fuel cells and for direct borohydride fuel cells. J. Power Sources.

[4] Elder J.P. (1962). Hydrogen ionization in the anodic oxidation of the borohydride ion. Electrochim. Acta.

[5] Indig M.E., Snyder R.N. (1962). Sodium borohydride, an interesting anodic fuel. J. Electrochem. Soc..

[6] Gardiner J.A., Collat J.W. (1965). Kinetics of the stepwise hydrolysis of tetrahydroborate ion. J. Am. Chem. Soc..

[7] Morris J.H., Gysing H.J., Reed D. (1985). Electrochemistry of boron compounds. Chem. Rev..

[8] Amendola S.C., Onnerud P., Kelly M.T., Petillo P.J., Sharp-Goldman S.L., Binder M.J. (1999). A novel high power density borohydride-air cell. J. Power Sources.

[9] Liu B.H., Li Z.P., Suda S.J. (2003). Anodic oxidation of alkali borohydrides catalyzed by nickel. J. Electrochem. Soc..

[10] Liu B.H., Li Z.P., Suda S. (2004). Electrocatalysts for the anodic oxidation of borohydrides. Electrochim. Acta.

[11] Gyenge E. (2004). Electrooxidation of borohydride on platinum and gold electrodes: Implications for direct borohydride fuel cells. Electrochim. Acta.

[12] Martins J.I., Nunes M.C. (2008). Comparison of the electrochemical oxidation of borohydride and dimethylamine borane on platinum electrodes: Implication for direct fuel cells. J. Power Sources.

[13] Ma J., Choudhury N.A., Sahai Y. (2010). A comprehensive review of direct borohydride fuel cells. Renew. Sustain. Energy Rev..

[14] Lima F.B.H., Pasqualeti A.M., Molina Concha B.M., Chatenet M., Ticianelli E.A. (2012). Borohydride electrooxidation on Au and Pt electrodes. Electrochim. Acta.

[15] Merino-Jimenez I., Ponce de Leon C., Shah A.A., Walsh F.C. (2012). Developments in direct borohydride fuel cells and remaining challenges. J. Power Sources.

[16] Oshchepkov A.G., Braesch G., Rostamikia G., Bonnefont A., Janik M.J., Chatenet M., Savinova E.R. (2021). Insights into the borohydride electrooxidation reaction on metallic nickel from operando FTIRS, on-line DEMS and DFT. Electrochim. Acta.

[17] Xu C., Chen P., Hu B., Xiang Q., Cen Y., Hu B., Liu L., Liu Y., Yu D., Chen C. (2020). Porous nickel electrodes with controlled texture for the hydrogen evolution reaction and sodium borohydride electrooxidation. CrystEngComm.

[18] Gyenge E., Atwan M., Northwood D. (2006). Electrocatalysis of borohydride oxidation on colloidal Pt and Pt-alloys (Pt-Ir, Pt-Ni, and Pt-Au) and application for direct borohydride fuel cell anodes. J. Electrochem. Soc..

[19] Geng X.Y., Zhang H.M., Ye W., Ma Y.W., Zhong H.X. (2008). Ni–Pt/C as anode electrocatalyst for a direct borohydride fuel cell. J. Power Sources.

[20] Tegou A., Papadimitriou S., Mintsouli I., Armyanov S., Valova E., Kokkinidis G., Sotiropoulos S. (2011). Rotating disc electrode studies of borohydride oxidation at Pt and bimetallic Pt–Ni and Pt–Co electrodes. Catal. Today.

[21] Wang G.J., Gao Y.Z., Wang Z.B., Du C.Y., Wang J.J., Yin G.P. (2010). Investigation of PtNi/C anode electrocatalysts for direct borohydride fuel cell. J. Power Sources.

[22] Yi L., Hu B., Song Y., Wang X., Zou G., Yi W. (2011). Studies of electrochemical performance of carbon supported Pt–Cu nanoparticles as anode catalysts for direct borohydride–hydrogen peroxide fuel cell. J. Power Sources.

[23] Yi L., Liu L., Liu X., Wang X., Yi W., He P., Wang X. (2012). Carbon-supported Pt–Co nanoparticles as anode catalyst for direct borohydride-hydrogen peroxide fuel cell: Electrocatalysis and fuel cell performance. Int. J. Hydrog. Energy.

[24] Tamašauskaitė-Tamašiūnaitė L., Baronaitė A., Stankevičienė I., Vaičiūnienė J., Kondrotas R., Juškėnas R., Norkus E. (2014). Investigation of borohydride oxidation on graphene supported gold-copper nanocomposites. J. Electrochem. Soc..

[25] Yi L., Wei W., Zhao C., Yang C., Tian L., Liu J., Wang X. (2015). Electrochemical oxidation of sodium borohydride on carbon supported Pt-Zn nanoparticle bimetallic catalyst and its implications to direct borohydride-hydrogen peroxide fuel cell. Electrochim. Acta.

[26] Duan D., Liang J., Liu H., You X., Wei H., Wei G., Liu S. (2015). The effective carbon supported core–shell structure of Ni@Au catalysts for electro-oxidation of borohydride. Int. J. Hydrog. Energy.

[27] Yi L., Wei W., Zhao C., Tian L., Liu J., Wang X. (2015). Enhanced activity of Au–Fe/C anodic electrocatalyst for direct borohydride-hydrogen peroxide fuel cell. J. Power Sources.

[28] Hosseini M.G., Mahmoodi R. (2017). The comparison of direct borohydride-hydrogen peroxide fuel cell performance with membrane electrode assembly prepared by catalyst coated membrane method and catalyst coated gas diffusion layer method using Ni@Pt/C as anodic catalyst. Int. J. Hydrog. Energy.

[29] Yi Q., Zhang J., Chen A., Liu X., Xu G., Zhou Z. (2008). Activity of a novel titanium-supported bimetallic PtSn/Ti electrode for electrocatalytic oxidation of formic acid and methanol. J. Appl. Electrochem..

[30] Hassan H.B. (2009). Electrodeposited Pt and Pt-Sn nanoparticles on Ti as anodes for direct methanol fuel cells. J. Fuel Chem. Technol..

[31] Abe H., Matsumoto F., Alden L.R., Warren S.C., Abruña H.D., DiSalvo F.J. (2008). Electrocatalytic performance of fuel oxidation by Pt_3_Ti nanoparticles. J. Am. Chem. Soc..

[32] Shao Z.-G., Lin W.-F., Zhu F., Christensen P.A., Zhang H., Yi B. (2006). A tubular direct methanol fuel cell with Ti mesh anode. J. Power Sources.

[33] Yu E.H., Scott K. (2004). Direct methanol alkaline fuel cell with catalysed metal mesh anodes. Electrochem. Commun..

[34] Freitas R.G., Santos M.C., Oliveira R.T.S., Bulhões L.O.S., Pereira E.C. (2006). Methanol and ethanol electroxidation using Pt electrodes prepared by the polymeric precursor method. J. Power Sources.

[35] Ding E., More K.L., He T. (2008). Preparation and characterization of carbon-supported PtTi alloy electrocatalysts. J. Power Sources.

[36] Brankovic S.R., McBreen J., Adzic R.R. (2001). Spontaneous deposition of Pt on the Ru(0001) surface. J. Electroanal. Chem..

[37] Sasaki K., Wang J.X., Naohara H., Marinkovic N., More K., Inada H., Adzic R.R. (2010). Recent advances in platinum monolayer electrocatalysts for oxygen reduction reaction: Scale-up synthesis, structure and activity of Pt shells on Pd cores. Electrochim. Acta.

[38] Gokcen D., Bae S.-E., Brankovic S.R. (2010). Stoichiometry of Pt submonolayer deposition via surface-limited redox replacement reaction. J. Electrochem. Soc..

[39] Gokcen D., Bae S.-E., Brankovic S.R. (2011). Reaction kinetics of metal deposition via surface limited red-ox replacement of underpotentially deposited metal monolayers. Electrochim. Acta.

[40] Tegou A., Armyanov S., Valova E., Steenhaut O., Hubin A., Kokkinidis G., Sotiropoulos S. (2009). Mixed platinum–gold electrocatalysts for borohydride oxidation prepared by the galvanic replacement of nickel deposits. J. Electroanal. Chem..

[41] Vaškelis A., Stankevičienė I., Jagminienė A., Tamašauskaitė-Tamašiūnaitė L., Norkus E. (2008). The autocatalytic reduction of copper(II) by cobalt(II) in aqueous diethylenetriamine solutions studied by EQCM. J. Electroanal. Chem..

[42] Angerstein-Kozlowska H., Conway B.E., Sharp W.B.A. (1973). The real condition of electrochemically oxidized platinum surfaces: Part I. Resolution of component processes. J. Electroanal. Chem..

[43] Burke L.D., Ahern M.J.G., Ryan T.G. (1990). An investigation of the anodic behavior of copper and its anodically produced oxides in aqueous solutions of high pH. J. Electrochem. Soc..

[44] Brisard G.M., Rudnicki J.D., McLarnon F., Cairns E.J. (1995). Application of probe beam deflection to study the electrooxidation of copper in alkaline media. Electrochim. Acta.

[45] Heli H., Jafarian M., Mahjani M.G., Gobal F. (2004). Electro-oxidation of methanol on copper in alkaline solution. Electrochim. Acta.

[46] Pyun C.H., Park S.M. (1986). In situ spectroelectrochemical studies on anodic oxidation of copper in alkaline solution. J. Electrochem. Soc..

[47] Abd El Haleem S.M., Ateya B.G. (1981). Cyclic voltammetry of copper in sodium hydroxide solutions. J. Electroanal. Chem..

[48] Fleischmann M., Korinek K., Pletcher D. (1972). The kinetics and mechanism of the oxidation of amines and alcohols at oxide-covered nickel, silver, copper, and cobalt electrodes. J. Electrochem. Soc. Perkin Trans..

[49] Miller B. (1969). Split-ring disk study of the anodic processes at a copper electrode in alkaline solution. J. Electrochem. Soc..

[50] Mukerjee S., Srinivasan S., Soriaga M.P., McBreen J. (1995). Role of structural and electronic properties of Pt and Pt alloys on electrocatalysis of oxygen reduction: An in situ XANES and EXAFS investigation. J. Electrochem. Soc..

[51] Toda T., Igarashi H., Watanabe M. (1998). Role of electronic property of Pt and Pt alloys on electrocatalytic reduction of oxygen. J. Electrochem. Soc..

[52] Jaksic M.M. (2001). Hypo–hyper-d-electronic interactive nature of interionic synergism in catalysis and electrocatalysis for hydrogen reactions. Int. J. Hydrog. Energy.

[53] Stamenković V., Schmidt T.J., Ross P.N., Marković N.M. (2002). Surface composition effects in electrocatalysis:  Kinetics of oxygen reduction on well-defined Pt_3_Ni and Pt_3_Co alloy surfaces. J. Phys. Chem. B.

[54] Kitchin J.R., Khan N.A., Barteau M.A., Chen J.G., Yakshinskiy B., Madey T.E. (2003). Elucidation of the active surface and origin of the weak metal–hydrogen bond on Ni/Pt(1 1 1) bimetallic surfaces: A surface science and density functional theory study. Surf. Sci..

[55] Kitchin J.R., Nørskov J.K., Barteau M.A., Chen J.G. (2004). Modification of the surface electronic and chemical properties of Pt(111) by subsurface 3d transition metals. J. Chem. Phys..

[56] Greeley J., Mavrikakis M. (2004). Alloy catalysts designed from first principles. Nat. Mater..

[57] Greeley J., Mavrikakis M. (2006). Near-surface alloys for hydrogen fuel cell applications. Catal. Today.

[58] Vaškelis A., Norkus E., Stalnionienė I., Stalnionis G. (2004). Effect of the Cu electrode formation conditions and surface nano-scale roughness on formaldehyde anodic oxidation. Electrochim. Acta.

[59] Vaškelis A., Jačiauskienė J., Stalnionienė I., Norkus E. (2007). Accelerating effect of ammonia on electroless copper deposition in alkaline formaldehyde-containing solutions. J. Electroanal. Chem..

